# Creating Learning Environments Free of Violence in Special Education Through the Dialogic Model of Prevention and Resolution of Conflicts

**DOI:** 10.3389/fpsyg.2021.662831

**Published:** 2021-03-17

**Authors:** Elena Duque, Sara Carbonell, Lena de Botton, Esther Roca-Campos

**Affiliations:** ^1^Department of Theory and History of Education, University of Barcelona, Barcelona, Spain; ^2^Faculty of Education, University of Girona, Girona, Spain; ^3^Department of Sociology, University of Barcelona, Barcelona, Spain; ^4^Department of Comparative Education and Education History, University of Valencia, Valencia, Spain

**Keywords:** special education needs, prevention of bullying, dialogic model of prevention and resolution of conflicts, inclusion, Zero Violence Brave Club

## Abstract

Violence suffered by children is a violation of human rights and a global health problem. Children with disabilities are especially vulnerable to violence in the school environment, which has a negative impact on their well-being and health. Students with disabilities educated in special schools have, in addition, more reduced experiences of interaction that may reduce both their opportunities for learning and for building protective social networks of support. This study analyses the transference of evidence-based actions to prevent violence in schools – the dialogic model of prevention and resolution of conflicts (DMPRC) – in the context of a special school, and its impact on the reduction of violence, the creation of egalitarian relationships, and the prevention of bullying. A case study with a communicative approach was conducted including in-depth interviews and communicative focus groups with the diverse participants to analyze the process of transformation carried out in the school and the main actions that give students a voice in the management and creation of egalitarian non-violent relationships. The results show that the inclusion of the students’ voices in the resolution and prevention of conflicts reduces violence, empowers special education students, strengthens friendship relationships, caring behavior, and active positioning among the community. The positive impact of the transference of the DMPRC to special schools contributes to students’ well-being and healthy development by offering safe and protective educational spaces and quality emotional education, also contributing to the achievement of the Sustainable Development Goals related to the elimination of all forms of violence in childhood.

## Introduction

School violence is a global problem affecting millions of children worldwide (Smith, 2002; Liang et al., 2007; Khoury-Kassabri et al., 2009; Murray-Harvey and Slee, 2010; Chen and Astor, 2012; Robers et al., 2012; UNICEF, 2014; Jiménez, 2019; UNESCO, 2019; Giavrimis, 2020). While the UN in the Convention on the Rights of Persons with Disabilities (United Nations, 2007) reaffirmed the international commitment to provide quality and inclusive primary and secondary education on an equal basis with others, much research shows that the most vulnerable groups, such as students with disabilities, are at greater risk of violence in mainstream schools (Reiter et al., 2007; Sentenac et al., 2011; Devries et al., 2014; European Parliament, 2015; Malecki et al., 2020).

Research shows the high rates of intimidation and harassment that students with disabilities suffer and the barriers which leave them defenseless and unprotected. The data show that they are more likely to be abused than non-disabled students (Malecki et al., 2020). Some research even suggests that they are three to four times more likely to be bullied than typically developing students (Devries et al., 2014). On the other hand, research shows that students with disabilities have fewer social ties and support networks, which leaves them more exposed to attacks. In this regard, barriers to reducing or eliminating violence against children with disabilities in mainstream schools have been identified, which increase the likelihood that these students will become victims of school violence. These barriers include the lack of a strong social network, rejection by peers and difficulties in relating with others (Méndez et al., 2017), having poor communication skills or personal characteristics that differentiate them from the others (CERMI, 2017), restrictions in school participation (Sentenac et al., 2011) and the limitations in adaptive behaviors, social skills and daily practices that intellectual disability entails (APA, 2013; Olivier et al., 2020). There is less research studying students’ vulnerability to violence in special education settings (Glumbić and Žunić-Pavlović, 2010), so this topic of study is still under-explored.

The consequences of violence for students with disabilities are being studied. It has been shown that students with disabilities are more likely to be victims and to have depressive symptoms due to this victimization (Olivier et al., 2020). According to research with autistic students, they are highly concerned about the possibility of being victims and the reported rates of bullying suggest that they may be at disproportionate risk of psychological harm (Ashburner et al., 2019; Gomes et al., 2020). When families in this study are asked about their concerns about the negative consequences of bullying on their children, the answers also include, among others, mental health issues, such as self-esteem, mental health, social participation, school attendance, academic performance, and behavior. Other studies continue to show that disability is associated with poorer mental health in adolescence, and that this fact is mediated by the bullying these adolescents experience. Thus, we could say that there is a harmful link between disability and mental health that seems to operate through bullying (King et al., 2018).

Therefore, it is urgent to know and implement effective interventions that can reduce the adverse effects of violence on this student body. Scientific literature shows that there is already evidence of successful interventions that have managed to reduce violence in schools (Berkowitz, 2014; Ríos-González et al., 2019). The most effective programmes so far to reduce violence towards disabled and non-disabled students, implemented in mainstream schools, coincide in enhancing peer intervention, support, friendship, and active positioning networks, as this is one of the most proved prevention factors (Bourke and Burgman, 2010; Rose et al., 2015; Hamby et al., 2016; Cook et al., 2017; Iotti et al., 2019; Clark et al., 2020; Iñiguez-Berrozpe et al., 2021). These programmes take into account the potential of bystanders to minimize or avoid damage. Evidence has shown that when programmes encourage bystanders to support or act on violence, it is possible to reduce and stop it (Banyard et al., 2004; Banyard, 2008; Swearer et al., 2010; Rose et al., 2015; Cook et al., 2017). Bystander’s motivations for intervening or not intervening in violence have also been well studied. Factors that have been shown to favor witness action to stop violence include clear school anti-violence policies, teacher and peer support for those who act and for those that create safe environments, and support networks that protect when intervening on behalf of victims by preventing attacks (Thornberg et al., 2012; Howe et al., 2013; Berkowitz, 2014). Some findings suggest that there is a need to develop intervention programmes that improve the school climate, promote trusting relationships between students and teachers and remove communication barriers to increase teachers’ awareness of school violence (Osher et al., 2012), as well as the feeling of belonging to the school (Syvertsen et al., 2009). Other research has analyzed aggressors’ motivations for violence, demonstrating, for example, that a motivation for violence is the pursuit of power and status (Saarento and Salmivalli, 2015), so interventions that aim to break the link between violence and higher social status will be effective.

Traditionally, there has been a tendency to approach the coexistence in special education schools or classrooms in a more disciplinary way and from a more behavioral perspective, rather than from a social or dialogic one (Beam and Mueller, 2016). However, there is increasing evidence of the relevance of interactions in the learning of children with disabilities (García-Carrión et al., 2018; Fernández-Villardon et al., 2020), which play a key role in the development of both cognitive abilities and positive feelings, such as solidarity and friendship. But more research is needed to understand how this interaction-based learning can affect the prevention of violence with students with disabilities.

The dialogic model of prevention and resolution of conflicts (hereinafter DMPRC; Villarejo-Carballido et al., 2019) is an action based on dialogue and the intervention of the entire educational community, promoting active positioning and solidarity and protective networks in the face of any attack. This model aims at overcoming the dominant socialization that links attraction and violence and achieves the building of more egalitarian relationships that combine the desire for the best values and feelings and prevents violence among peers (Gómez, 2015; Navarro et al., 2018; Puigvert et al., 2019; Elboj-Saso et al., 2020; López de Aguileta et al., 2020; Torras-Gómez et al., 2020). This study aims to investigate whether the measures that have been proved to be effective to prevent violence among students in ordinary schools can work in special schools, since social links and support networks may be more limited by the very difficulties of interaction among students and by the more individualized work that is usually carried out in this type of school. More specifically, this contribution analyses how the DMPRC has been transferred to a special school and what its impact has been on the prevention of violence in students with disabilities.

## Materials and Methods

A case study has been carried out in which the voices of participant students and teachers have been included, contributing to the collective creation of knowledge according to the premises of the communicative methodology (Gómez et al., 2011). This methodology builds on the dialogue that is created with the end-users of the research, which includes their voices in an egalitarian dialogue to jointly build knowledge that enables a deeper and more accurate understanding of the reality under study, achieving the objective of social impact which is the transformation of such reality (Gómez et al., 2012). Several studies have demonstrated the adequacy of this methodology to conduct research regarding vulnerable groups (Puigvert et al., 2012; Gómez et al., 2019), especially when there is the objective of achieving social impact.

Following this methodology, in our study we ensured that the data collection techniques not only allowed gathering the end-users’ narratives and perceptions, but also that dialogue was at the center of the process, in order to discuss with the participants their experiences as well as the existing evidence on the topic so far. In this way, we could identify the exclusionary components of reality –which refer to the barriers and difficulties that people with disabilities encounter to overcome the risk of being bullying victims, and the transformative components – those that contribute to overcome such barriers. This methodology allows, through dialogue with the participants, an agreement on these exclusionary and transformative components, which enhances the validity of the results and strengthens its potential social impact.

### Case Study

The special school that is the subject of this case study is a school that has been implementing, since the 2013–2014 academic year, successful educational actions identified in the INCLUD-ED research project of the 6th European Framework Programme, which have already demonstrated have a positive impact on students with special educational needs (García-Carrión et al., 2018). The school serves students from seven Valencian municipalities and currently has 160 students between the ages of 3 and 21, who attend preschool, primary and secondary education, the transition to adult life program, and other training programmes.

In the academic year 2016–2017, they began to apply the DMPRC, starting initially in some classrooms until it became the school approach to improve coexistence. The DMPRC is a successful educational action based on the theory of preventive socialization of gender violence and the scientific theories that emphasize two key aspects to improve education: quality interactions and community participation. This action is grounded on promoting dialogue within the community as a means to create the coexistence rules of the school-based on consensus, usually through assemblies. In this regard, the DMPRC is developed in the framework of the dialogic learning (Flecha, 2000). This is a communicative perspective of learning that understands that people learn through dialogue, and through dialogue transformations can be done in the interpersonal relationships and in the environment. In the special school, assemblies are held in the classrooms and with the classrooms representatives to include the students in the dialogic process of improving coexistence. Within this dialogic model for coexistence improvement, there is a specific action called the Zero Violence Brave Club, which was created as a strategy that helped teachers in other schools put in practice research evidence on the benefits of bystander intervention, uniting the language of ethics and the language of desire, the creation of support networks and reaching consensus on clear rules of zero tolerance to violence, all this mediated by dialogue among community members. With the aim of using a concept known in the community, in the special school they use the name of the Zero Violence Brave Club when they refer to the MDPC, as the students feel identified with it and is attractive for them.

### Data Collection and Analysis

The following techniques were used to collect information: (1) in-depth interview with a primary and a secondary school teacher, (2) focus groups with students from different educational stages, and (3) an evidence record table which was delivered to the school in the academic year 2018–2019 to collect relevant data on the implementation of the DMPRC (see Table 1). Due to the pandemic situation, the fieldwork was carried out taking into account all the safety measures and following the procedures agreed with the school. Following the communicative methodology, the guidelines for the data collection techniques were developed jointly in a meeting with two teachers to discuss the content of the data collection and the language to be used in the focus groups with the students. The meeting was held with these teachers because they have been involved in the implementation of the DMPRC from the beginning and because they are the ones who have worked with the students who were going to participate in the focus groups and therefore know first-hand the language that they use and how the DMPRC is carried out. With the aim of gathering the maximum number of voices, a total of eight teachers from the school held two meetings (the first involved four primary school teachers and the second four secondary school teachers and teachers from the transition to adult life program) to discuss and dialogue the questions and evidence that were going to be dealt with in the in-depth interview. The conclusions that emerged were collected by two of the teachers, who subsequently participated in the in-depth interview.

**Table 1:** Data collection techniques.

Interview	One primary education teacher
	One secondary education teacher
Focus groups	One with primary and secondary education students
	One with students of the transition to adult life program and of the gardening training program
Evidence record table	Information on the implementation of the DMPRC

To ensure the anonymity of both teachers and students, each participant was assigned a code which was used in the transcription and analysis of the data. Before the fieldwork was carried out, the participants were informed of the objective of the study, of the anonymous and voluntary participation and that the data would be treated confidentially and used only for research purposes. All participants agreed to provide researchers with information relevant to the purpose of the study and signed an informed consent form. Family members of the minors also signed the informed consent. The ethical requirements were addressed following the Ethical Review Procedure established by the European Commission (2013) for EU research, the Data Protection Directive 95/46/EC and the Charter of Fundamental Rights of the European Union (2000/C 364/01). The study was fully approved by the Ethics Board of the Community of Researchers on Excellence for All (CREA) with the number 173 20210117.

#### Communicative Focus Group

A total of two focus group sessions were held throughout the case study with school students; besides the researcher, the students’ teachers were also present to facilitate communication. Seven students participated in the CFG, who had the following disabilities: pervasive development disorder (two cases), autism, moderate intellectual disability (three cases, one of them with a language disorder), and severe intellectual disability. In the first CFG with students, three students (two from primary education and one from secondary education) aged 9, 10, and 12, respectively, participated. In the second CFG with students, four students participated, three of them were in the transition to adult life program and one in the gardening training program, aged 17, 18, 19, and 20, respectively. The themes proposed for the dialogue were accompanied by evidence from previous research on the topic (violence prevention and the factors that protect from bullying), which was shared by the researchers with the participants to contrast it with their own experiences. These themes revolved around the importance of friendship to prevent violence, the need to break the silence by taking a stand against violence, the importance of seeking help and protecting those who need it, the existence and knowledge of clear rules of zero tolerance for violence and the creation of dialogic spaces in which to denounce violence and feel supported in order to increase the perception of safety and well-being. The teachers of primary and secondary education collaborated in the writing of the questions in order to facilitate the understanding of the language and worked previously with the students to ensure their comprehension. With the students in the transition to adult life program and in the gardening program, the teachers prepared the interviews in dialogue and made it possible for two students to write down their answers to give them more confidence. The teachers stayed in the two CFGs with students taking the role of communication facilitator. Sometimes they reformulated the questions using the same language they use in the classroom, and sometimes they repeated the ideas expressed by the students and added language to the gestures they used in communication. This help has been a key issue to carry out the interviews with the students and to be able to overcome the barriers in communication by making it possible to incorporate the students’ voices in the research process and in the creation of the results. The CFGs were audio recorded with the prior consent of the students’ families and transcribed for later analysis. These aspects of communication were taken into account in the transcription of the interviews along with the voices of the students and teachers. Two aspects need to be clarified: the first one is that when students refer to the DMPRC, they do so by talking about the “Zero Violence Brave Club,” as it is usually called at school, and the second one is that, in the results section, when the students’ voices are reported, they are included in a dialogue with their teachers in order to reflect, as faithfully as possible, the importance of the scaffolding in the interview.

#### Interviews With a Communicative Approach

An in-depth interview was conducted with two teachers (one primary education teacher and one secondary education teacher). The objective of the interview was to learn about the process of implementation and impact of the DMPRC in the school. One of the main topics discussed in the in-depth interview with the teachers was the steps they took to implement the DMPRC from the beginning to the present day, to analyze how they made the transference of this approach to the improvement of coexistence and the prevention of conflicts to the special school, which up to that moment had only been implemented in mainstream schools. The rest of the topics revolved around the impact that the implementation of the DMPRC was having in the school on violence prevention, on the inclusion of students’ voices in the creation of more egalitarian relationships and quality friendships, on the increase in complaints about violence and on the empowerment of students themselves, on the creation of support networks that protect them, and on the effect that this has had on their well-being and happiness. This interview also included the debates and contributions that arose in the two previous internal meetings between the school’s teaching staff. These interviews were conducted in person, audio recorded ensuring the Covid-19 safety measures, and subsequently transcribed.

#### Evidence Record Table

An evidence record table was facilitated to the school in the academic year 2018–2019 with the aim of gathering evidence of the impact that the implementation of the DMPRC was having on students, including narratives, statements, or examples of situations. Therefore, the data collected were qualitative. The evidence record table was used by the teachers who implemented the DMPRC, who made a written record relevant information that evidenced how the DMPRC was contributing to prevent and overcome violence. The excerpts included in the results section are identified as “evidence record table.”

## Results

The analysis of the evidence collected shows four main results. The first one is the possibility of successfully transferring the DMPRC in a special school. The second one is the impact of the application of DMPRC on improving the school climate and coexistence, emphasizing the strength of this approach as a preventive measure against violence. The third result is the increase in complaints thanks to the empowerment of students who feel listened to and supported, and the fourth result is the increase in the creation of support and friendship networks that can act as a protective shield against any attack and improve their well-being.

### Transferability of the Dialogic Model of Conflict Prevention and Resolution to a Special School

Before starting to promote the DMPRC, this special school began to introduce the Successful Educational Actions in a phased manner. They first launched Literary Dialogic Gatherings and later they applied the DMPRC, which is being carried out from the 2016–2017 school year. The first proposal emerged from the management team and started to be implemented in some classrooms. Progressively, more teachers joined in, motivating each other until it became a line of action in the overall school that is present in the day to day. The teachers we interviewed highlight two steps that were key to its implementation. The first one is the internal training among the teachers themselves. Those with more years of experience explained to those who have just arrived or wish to incorporate the DMPRC how they have done it and the results obtained. The second step is the creation of a commission to promote and accompany implementation at each educational stage.

It may be that there are people who do not know how to do this and when they see that it works and gives good results they get hooked. Creating this commission, so that it flows … I came together to explain how I did it … in this commission last year they created a kind of script summary of these actions of how to carry them out in each educational stage (Primary education teacher, interview).

The implementation in primary education, secondary education, and in the transition to adult life program varies due to the age of the students, but the changes are not substantial to the DMPRC itself, instead, they have to do with the presentation of the stories, the vocabulary, or the more visual aids. They refer to strategies teachers have introduced to make it easier for students with disabilities to understand the rules or internalize them. As follows we describe the differences in the implementation of the DMPRC in each educational stage and the actions that are carried out in the same way.

In primary education, each school year begins with the reading of the story “Zero Violence Brave Club.” A teacher explained that if children cannot read, it is the teacher who reads the story, and all the children comment on it afterwards. Afterwards, in the assemblies held first thing in the morning, the behaviors considered to be correct or incorrect for the group are agreed upon. Later on, the class decides whether to have a Zero Violence Brave Club and a special space is created within the classroom for this purpose, in which each child chooses a superhero and puts his or her face on it. In another assembly in the morning, the rules for the prevention of violence were discussed. As the teacher explained, at the beginning, rules were not so much linked to the prevention of violence, but they had more to do with classroom rules. Little by little they realized that it was very important that they were exclusively related to the prevention of violence. The norms are agreed upon by all and are changed every year according to what they consider important, although they recognize that there are some rules that are maintained every year. They explained that it is important to formulate them in a positive way so that they are more effective for children and thus reduce the chances of them skipping. The rules are discussed in order to facilitate their deep understanding and to collectively construct their meaning.

(…) if the rule is to treat us well, what is it, to say nice words to us, to help and take care of our friends? Another rule may be to respect the body and, depending on the characteristics of the students of that year, we may or may not add more. The rules become more specific. For example, if I go to the toilet, I close the door because it is my body, it is my privacy, when I want to give a hug, I will ask … he will give me a hug, if I say no, you must respect it (Primary education teacher, interview).

Some of the rules that are usually maintained year after year are: treat well each other, respect each other’s body and tell the truth. These rules are applied everywhere in the school, not just the classroom. Before going out into the playground, the rules are reminded every day, so that they are kept in mind and not forgotten. Teachers remind them of what they can do to ensure that the playground is a safe space by encouraging them to take an active stance against violence. They do this by using phrases that they repeat every day.

(…) when we go out into the playground we say: eyes wide open, watch out for a cowardly attitude, make a magic curtain, or protect someone (Primary education teacher, interview).

When they speak of a cowardly attitude, they mean behavior that includes violence. This concept helps to make such violent behavior unattractive by making it easier for courageous behavior that excludes violence to become attractive. The magic curtain means not paying attention to those who do not behave courageously, but instead giving attention to those who are victims or who act courageously by denouncing or protecting. People who do not use violence are valued socially, giving them a lot of appeal.

Another important moment for the DMPRC is the classroom assemblies. These are held three times a day, in the morning, after the playground, and in the afternoon. In the first one, the agreed-upon rules are remembered every day, people who are in the club of the brave are named and they sing the song that reminds them what to do if they are getting nervous. They are also reminded of a message that helps them to be group conscious and reinforces group cohesion: “we do group together” (Primary education teacher, interview). In the second assembly, they are asked what they have played, with whom, if they have been brave and if they have seen a cowardly attitude encouraging them to share what they need. In the third assembly, the same questions are asked again, encouraging dialogue. The success of these spaces for dialogue is due to the strength of the group, which rejects violent behavior and decides together when someone leaves the Zero Violence Brave Club with arguments of validity.

When someone is having cowardly behavior, we all decide together when we are going to talk about it again, that is to say, we decide how long he will have to show us that he treats us well. If for example, it has been during the recess time, we try not talk about it again before another recess time has passed. It is agreed between all of us and they know that you have to show until that moment that you are brave enough to go back in (Primary education teacher, interview).

At this age, they place great importance on repetition and rehearsal of situations that “train” them so that when faced with a situation they know how to recognize violence and how to respond to it by keeping themselves safe. This helps students who have more difficulties in reasoning or reflect consciously.

(…) for some of the students reflection is more complicated, but if you rehearse it many times … that when they touch you, you say “stop” … when they are in the playground and they are touched, they will say “I don’t like it.” Modeling gives them the ability. Repetition helps to assimilate even if they haven’t done as much thinking (Primary education teacher, interview).

In secondary education and the transition to adult life program, the implementation of the DMPRC started directly with the consensus of the rules in the classrooms, putting them in positive to avoid challenging behaviors in the face of the rules in negative. In the dialogues on the norms to prevent violence, much thought is given to whether they will really serve this purpose or not. At this educational stage, the Zero Violence Brave Club is also made but no superheroes are chosen. Instead, in the classroom space dedicated to the club, their names used following the same dynamic as in primary education. Through the dialogues, they seek a consensus about what it is to be brave and about what attitudes they are not going to allow. In the language used, courageous behavior and active positioning are made attractive.

In the first moments, they did the Zero Violence Brave Club with some supports such as a point system for the students who presented many challenging, negative and aggressive behaviors.

(…) I have reduced this visual aid over time because they are now able to know who has had a cowardly attitude, they remember … this year in the classroom I have had to make an adaptation, because we have needed the panel, I have made a passport of the brave: what we intend to fulfill and when we do not fulfill it we do not get the stamps (Secondary education teacher, interview).

These adaptations are superficial and most of the time temporary and do not affect the basis of the DMPRC or that of the Zero Violence Brave Club, which are: egalitarian dialogue in the process of consensus on norms, attitudes of active positioning in the face of violence, the desire for non-violence, and the creation of support networks. In this sense, one teacher explained that something she considers fundamental for the good functioning of the assemblies is the previous work they had done on the seven principles of dialogic learning because it has allowed them to train egalitarian dialogue, which is what makes it possible for them to move away from power relationships. They stress the importance of remembering every year what egalitarian dialogue is, because it is very important for these students to go deeper into this concept little by little.

(…) working on the seven principles^1^ has been fundamental, that they understand what they mean, because in the end we base our action on egalitarian dialogue and it is no use for us to hold an assembly if it is not present (Secondary education teacher, interview).

All the instructions that are agreed upon, the rules that are discussed and reflected upon, constantly remind them of who they want to be as a group and as individuals, and being able to do so in a group with honesty helps them to walk towards that transformation. In addition, teachers also experience this transformation by being part of this collective dream.

It is a transformation that has taken place over the years, and I have experienced it in this way. We have gone from a very different education on these issues to starting with the MDCP and transforming ourselves with them. I think we have transformed with them and they also realize that we have changed too, and they demand that others change too and join the club (Secondary education teacher, interview).

The dialogic contexts that are created in the school, where the strength of the group is the motor of change, is an opportunity to gain confidence and to progressively be able to transfer it to other contexts. It has not been possible to confirm the effectiveness of the transfer outside the school because the contexts are different and are not always conducive to quality interactions that value the courage of good treatment and action in the face of violence. An example of the difficulty that some students have in transferring the Zero Violence Brave Club outside the school is shown in this narrative by a teacher in the interview.

It depends on the group he is in because sometimes we have had experiences … like that of a former student who told us: “they threw a stone at me and I said I’m not going to allow it anymore” and she was told “that’s children’s stuff” and the girl said: “well, I’m not going to school anymore.” This happened in a basic qualification programme. We told her to try … and she said: “in my school before, they listened to me and what I said was important” (Secondary education teacher, interview).

### Impact on Violence Prevention and Improvement of School Climate

Having a model of coexistence that is based on prevention is crucial, because violent behavior can appear and increase rapidly towards an escalation of violence, according to the teachers in the interview. Detecting such behaviors even before they appear and teaching the group to detect them by having clear rules that help to stop them or even that they do not start, is crucial to improve the climate of the classroom and the school and to promote safer environments where learning is not altered by violence. Working in advance with special needs students is very important and not always easy. The Zero Violence Brave Club helps them to anticipate negative and violent behavior, but not only from adults, but also from peers. Enabling students to take an active stand against violence allows them to train this approach.

In the interviews with students, they told us that when they saw that a classmate was becoming nervous and could trigger violent behavior, they initiated a dialogic interaction to anticipate violent behavior and redirect that reaction towards non-violent behavior. Thanks to the DMPRC, they have learned strategies that they use with their colleagues with the aim, as they say, that no one leaves the club, that is, that no one engages in violent behavior. They help each other to self-regulate their behavior through dialogic interaction, achieving the effect of anticipation and prevention.

If someone is brave and is getting a little nervous, what do we say to help them? (Primary education teacher, interview).

Think, talk, what’s wrong, can I help you? 1, 2, 3, 4, 5, 6, 7, 8, 9, 10, I know how to relax (Primary education student 1, CFG).

To make this possible, the teachers report that in class assemblies they continually dialogue and remember words and gestures that they can use as strategies when they see someone getting nervous (the prelude to aggressive behavior). The difference with the previous model of coexistence, more horizontal and less social, in which only the teacher acted, lies in the fact that now the whole group (teachers and students) intervene, and prevention in strengthened. This is what we have called in the previous section the “strength of the group.”

(…) for example, when someone comes up to me with a cowardly attitude, we see that they’re not going to respect my body or treat me well, then the “stop” or “I don’t like it” before he pulls my hair … if we see them coming I say “stop, I don’t like it,” that’s one of the strategies. Another one that we use a lot is “think, talk, what’s wrong with you.” For example, someone has fallen to the ground and we see that they are nervous and start with a little kick … accompanied by the gesture we tell them: “think, talk, what’s wrong with you, can we help you?” And they repeat this a lot. And we sing a little song about relaxing too (Primary education teacher, interview).

The collective verbalizing of these phrases in a repeated way helps the students who have more problems with internalization to achieve this over time. The type of phrases are: “no is no,” “think, talk, what’s wrong with you” or “we are making a curtain.” When someone has broken the rule and has acted violently, this phrase is used: “we are not going to allow you to do this, you are going to leave alone, you are not going to be our friend.” With this type of agreed rules, they are continually reminded that the group rejects violence and that to be part of it, they have to treat everyone well.

At the beginning, these actions were very much directed by the teachers until students have internalized them and, at this time, they are spontaneous and are generalized to other spaces such as the playground and the school canteen.

When we are in class and there is a student who gets nervous and it is very likely that the behavior will appear, the students redirect the situation in anticipation of violent behavior appearing (Evidence record table).

So that prevention can go beyond the classroom, a panel has been placed in the playground where all the students who have the Zero Violence Brave Club in class are displayed. If someone has left the club because of violent behavior they have had in the classroom, they also leave the panel in the playground so that the strength of the community is greater. In this way, all students can consciously choose whether or not to play with someone who has behaved violently. On the one hand, it encourages freedom of choice when looking for playmates, and on the other hand, the rejection of violence by the whole school gains strength. It is no longer just in the classroom, but when a playmate is not treated well, the rejection is collective.

(…) so that a larger support network is created, one that is not only of the class … and that the teachers themselves can be more successful when they encourage others to go and play with those who are alone … (Primary education teacher, interview).

Some improvements observed by the teaching staff stand out. Some of these are: a much calmer classroom environment, dialogue on how students’ relationships are or how they treat each other, the avoidance of escalation of violence with a reduction in the levels of violence, an increase in the level of trust and the support, protection and accompaniment of students, and a reduction in the number of disciplinary measures.

(…) the brutal impact is to reduce violent attitudes, aggressions (Secondary education teacher, interview).

(…) in the classroom there is a much calmer atmosphere, things are talked about, we say “we are your friends, we want to help you” before everything starts to blow up, before there is a lot of shouting … it is a more trusting space (…) the coexistence committee checks the behavior reports and there are less (Primary education teacher, interview).

The students at the transition to adult life program say that this year they are safer because there is a better climate in the school, i.e., they notice that the atmosphere has improved. They associate it with the creation of more respectful relationships between everyone and with the improvement in the behavior of the students. When the feeling of safety increases, it is an indicator of improved coexistence and a decrease in violence.

Yes, because of the Zero Violence Brave Club, I am happier this year, more so than in other years because of the Zero Violence Brave Club. I have seen that it is better, I see it better than other years, because people who did not behave well, this year they respect the rules more and behave better, I feel safer with the people, because of the people who are around me … I feel safer, above all there is more respect (Transition to adult life program student 3, CFG).

An example of how the DMPRC has reduced the seriousness of violent behavior is shown in the story of a primary school teacher who reported the case of a student who was in the center. He was a student who displayed very violent behavior such as hitting, pinching, kicking, or biting. For him, a rule was prioritized in the club of the brave that was “treat well.” From then on, they decided to start with one of the violent behaviors he presented to make it disappear, the one chosen was pinching. Every time he pinched, he left the club. When the behavior is not very internalized, a very specific consequence is added, which can be, for example, that they are left without 5 min of recess time or they do not listen to their favorite song, because at first these students finds it difficult to feel part of the group and until the social aspect has an impact, it is accompanied by this type of measures. It is a strategy that can last for some time until this consequence disappears, leaving only the social consequence, which is what really has the strength. Little by little the rules they have to meet are becoming more demanding.

**Figure 1:**
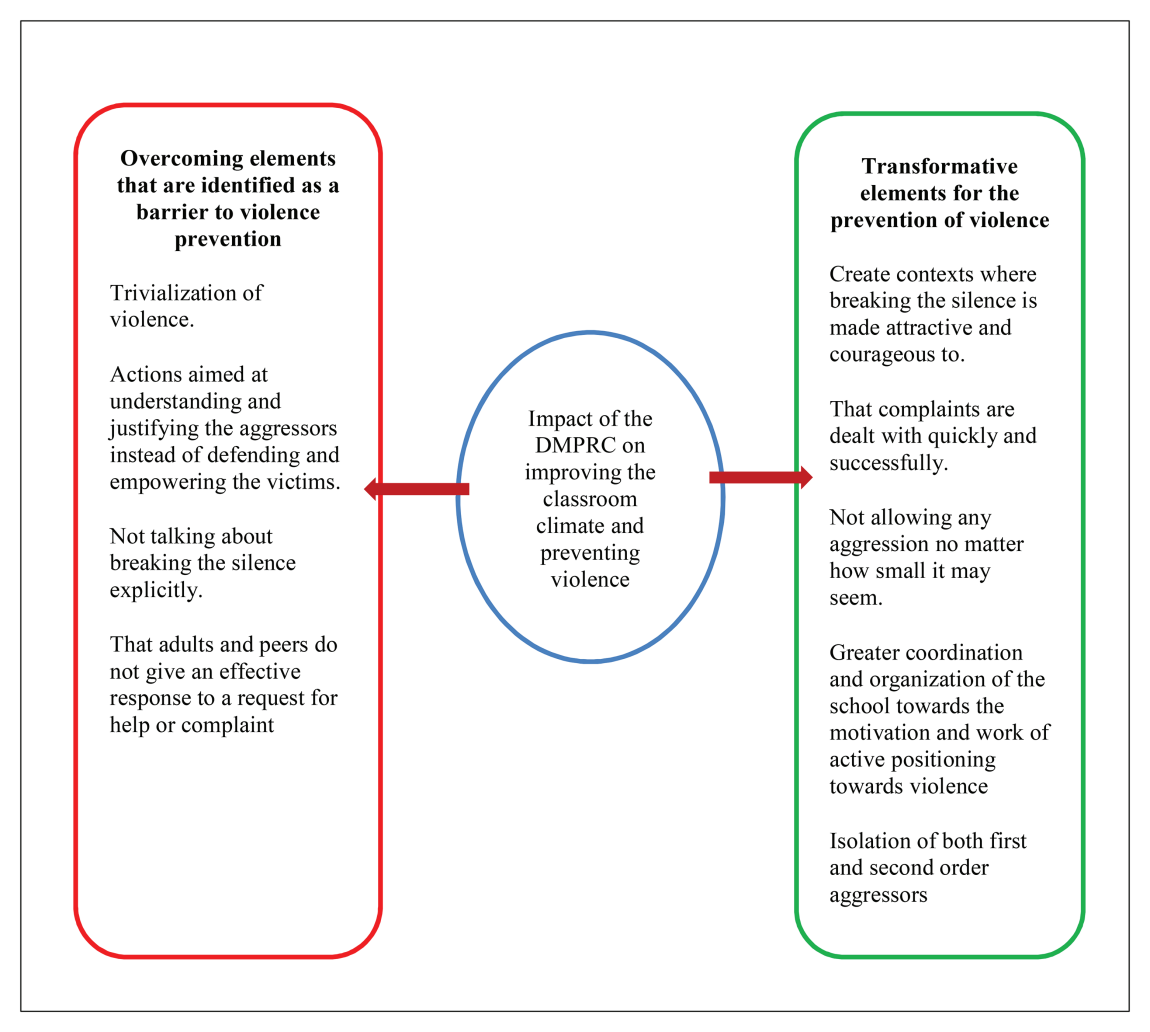
Impact of the dialogic model of prevention and resolution of conflicts (DMPRC) on prevention and improvement of coexistence: barriers that it overcomes and transformations that it achieves.

Finally, in this section, we would like to highlight two impacts on the prevention of violence which have been extracted from the evidence record table. The first refers to how, through the DMPRC, some elements that have been identified as barriers to violence prevention have been overcome, and the second has to do with the transformative elements that have appeared. Figure 1 shows on the left the elements identified as barriers that have been successfully overcome and, on the right, the transformative elements that have been achieved (see Figure 1).

### Impact of the Inclusion of the Students’ Voices: Students’ Empowerment and Increase in the Number of Complaints

The creation of interactive dialogic spaces where the voices of students with disabilities are heard and valued is one of the key aspects for the impact of the DMPRC. Within the classrooms, we have already reported the impact of the assemblies which are held three times a day, but, in addition, at the school level, there are also assemblies where the delegates of each class represent their classmates and their dialogues revolve around the desire for good treatment and the rejection of violence.

The voices of students with disabilities tend to be unheard and silenced, resulting in greater vulnerability to violence and reduced self-esteem and security. But the DMPRC makes their voices be raised and heard, turning these students into protagonists of their lives and relationships and providing them with the necessary empowerment to identify violence, denounce it, and reject it. According to the teachers, the students have gained a lot of confidence and one of the impacts is that they now have high expectations about the type of relationships they build, they are no longer satisfied with just any type of interaction.

The students are able to talk because they feel that they are being listened to, they demand high expectations on them. Many past issues of bullying in mainstream schools come out … situations of mistreatment in their home have come out, relationships issues … (Secondary education teacher, interview).

A primary education student said that he is now more attentive in the playground to see if someone is not treating others well, and if someone is treating someone violently, he identifies him more quickly and tells a teacher. One of the rules that has helped them to make this happen is “telling the truth” because it values the sincerity and courage of those who denounce an injustice or an aggression.

Do you remember any rules? (Primary education teacher, CFG)

Tell the truth. Eyes wide open in the playground in case we see a cowardly attitude (Primary education student 3, CFG)

And what do we do? (Primary education teacher, CFG)

I’m going to tell it to the teacher (Primary education student 3, CFG)

The impact of the DMPRC goes beyond the school, because when something has happened to them, the rules remind them that they have to tell it at home too.

Who are you going to tell if this happens? (Primary education teacher, CFG)

To the teacher (Primary education student 3, CFG)

And at home? (Primary education teacher, CFG)

To Mum and Dad (Primary education student 3, CFG)

And to the brothers and sisters (Primary education student 1, CFG)

Respect for the body is worked on continuously; it is another of their rules and the students are clear that if someone touches their body it is not a joke, nor a game and they have to report it, which contribute to prevent abuses.

And if someone touches our body, is it a joke, a game, or a secret? (Primary education teacher, CFG)

No (Primary education student 1, CFG)

A large number of students who attend secondary education have been previously enrolled in regular schools. Some of them tell their stories of suffering from bullying and not being listened to, and even say that if the DMPRC had been applied in the regular school, they might still be there.

Those who have been here since preschool have already started to work on this from a young age, but those who come from a mainstream school … most of them come with a story of bullying situations and say “why didn’t they do this in the other school?” We constantly experience this “if they did this in all the schools, I wouldn’t be here.” They also say “I don’t want to leave here because they listen to me, they value me, I can learn” (Secondary education teacher, interview).

The students at the transition to adult life program recognize that since they have had this dialogic model of coexistence at school, they are better and happier because they feel listened to, supported and safer. They also show happiness when they see themselves capable of sharing and giving away courage and true friendship to make other people safe and happy too.

If I feel better, yes, I feel that my colleagues are listening to me. It is an example, if someone ever messes with anyone, I stand in front of him and say, ssshh quiet, stop, leave him alone, because I will not allow any harm to come to him, as the good friend and delegate that I am (Transition to adult life program student 4, CFG).

A primary education teacher explained that with other more disciplinary models of coexistence the voices of these children are very much silenced. According to the teacher, this model in which their voices take on a central role transform the environment and allows them to feel empowered to dream of better relationships, to seek them out and ultimately to be happier.

Now they claim and demand respectful treatment, one primary education student started crying when I raised my voice to her. It was a way of telling me that she didn’t want to be treated like that (Primary education teacher, interview).

One student insists that he likes the Zero Violence Brave Club because it helps to ensure that everyone is treated equally, not only among the students but also from the teachers to the students. This breaks with the power relations that favor the law of silence and promotes dialogic and egalitarian environments that, on the contrary, encourage students to be able to denounce violence regardless of who is doing it.

That people treat you well, that people treat you well not only students, but also teachers or anyone else in this school, that there is respect, that we respect each other equally, for example, the physiotherapists who work with the children, the educators … everyone in general (Transition to adult life program student 3, CFG).

The suggestion box has helped them to be able to denounce situations in which they have not been treated well, whether they occur by a peer or an adult. Reporting that a teacher is not treating another teacher or student well is an act of courage that can only be done when the environment is safe and trustworthy, when reporting the situation is socially valued and when there are support networks to protect you from attacks.

In the suggestion box, they write things that need to be improved, for example, if they see a cowardly attitude from an adult towards a student … in class they feel that it is a safe environment, but if they are not able to verbalize it because they are a little afraid … they use the box. There is no such thing as a snitch, the message is “you are brave because you say things and take a stand.” They report “I saw a teacher who spoke badly to a classmate.” They put it in the suggestion box and we discuss it (Secondary education teacher, interview).

The confidence they have gained leads them to request assemblies to speak out and denounce the violent events that have taken place. This is a protective factor since one of the elements that has been identified as important in preventing and overcoming violence is the creation of safe spaces to break the silence, and with the DMPRC this is possible. Empowerment among peers as agents of change is detected, which has led them to report not only situations that happen in the school but also those that occur at home, in the park, or with neighbors.

In general, the teachers value very positively the increase in the number of complaints and the students’ self-confidence. They told us about the impact of the students’ participation in various conferences in which they have been able to listen to researchers talk about these issues and share their own success stories at roundtables, moving from being victims to being role models.

(…) the importance of increasing self-esteem, they feel capable of doing more, of speaking and having their voice heard, I have seen this when we have taken students to conferences, congresses where they have participated as listeners or speakers, and the personal satisfaction on their faces … Going to a conference and saying: “teacher, you’re not crazy, they say the same thing here as we do in class” and they tell it, “I’ve transformed myself,” “I used to attack when I didn’t like something and now I’m able to stand up and talk to my mother” … it has changed their lives completely (Secondary education teacher, interview).

### Impact on the Quality of Students’ Relationships and the Creation of Support and Friendship Networks

Traditionally, the coexistence in special schools tends to be approached in a more disciplinary way and from a more individual, behavioral and not so much social or dialogic perspective. The fact of dialoguing and reflecting together on what their relationships are like allows them to dream and seek quality relationships where violence has no place. The importance of group strength has already been reported, and it is clear the importance of support networks and friendships in preventing violence and encouraging reporting.

With the DMPRC, opportunities are created to show solidarity with victims, to protect them when they are exposed to an aggression and to denounce them if necessary. In the evidence record table, teachers explained how a student acted in the face of an aggression to protect the victim from a new aggression by giving her support.

A 9-year-old student was attacked by another student. A classmate who was in the playground observing the situation, approached the student who had been attacked, and asked him “what’s wrong, come and play with me,” shook his hand and took him out of the conflict situation, leaving the aggressor alone and going to play with other classmates (Evidence record table).

The shield is a strategy they use to protect other children and they accompany it with a gesture. The students are clear that they must make a shield for the weakest, the victim, and they do this by saying phrases such as “stop” or “I do not like it.” These support networks are crucial to reducing violence and its negative impact.

Does the Zero Violence Brave Club help you to protect yourselves more or to make a shield? (Researcher, CFG)

Yes, we do it to the brave, to those who are not treated well (Primary education student 1, CFG)

When they don’t treat well, what do we do? (Primary education teacher, CFG)

“No, stop, I don’t like it” (Primary education student 3, CFG)

And we make him a shield together (Primary education teacher, CFG)

The message that “friends are the ones who treat you well” is very much emphasized and this helps them to choose their friendships with a criterion of good treatment. It also helps them to build higher-quality relationships with the friends they already have and to transform them from high expectations.

In primary education we work a lot on friendship, we have done the friendship workshop, little theatres … we have talked about what I agree or not with a friend … the radical change we saw was that two children who were close friends were able to say that one had been a coward. When it is a friend of theirs and they have to denounce a friend of theirs … it is what has been most difficult but we have seen it. Rejecting these behaviors also in people I love (Primary education teacher, interview).

The DMPRC has helped them to learn to be better friends; they identify friendship with people they treat well, and also with those they protect. They learn that friends are those who also tell you that you are not doing well. Another indicator of friendship that the students themselves relate is that a friend is the one you can dream about or the one who helps you to become a better person.

I feel good because I help by giving advice to the victim, we make a real friendship team and we all support each other between the two classes to improve and transform ourselves and, we help each other to try to say good things and make constructive criticism that makes the other feel good and helps to be a better person (Transition to adult life program student 2, CFG).

I help my friends in class, I am happy, we help together, we are happy, we have dreams, we are equal (Transition to adult life program student 1, CFG).

A secondary education teacher and the student who told that they were now dreaming together explained how he had become a better person thanks to the DMPRC. They said together that he used to be more nervous, did not use words and did not always treat people well, and that now he wants to be a brave person and help others to be brave too. The Zero Violence Brave Club has given them the opportunity to imagine themselves differently, being brave people who treat each other well and help others to achieve that same dream. Without the feeling of friendship in the background, this would not be possible.

One of the messages that the teachers now convey is that true friends are those who do not leave you alone in the face of aggression, they are those who make a shield for the person being attacked.

Every day we say that if something happens to them, they have to be brave and tell the truth, friends have to be attentive and we too, no one can be left alone (Teacher).

The teachers also recognize that they now give more importance to their role in creating support networks for victims, which violence is no longer minimized or normalized, that it is given importance, that it is rejected, and that those who receive it are supported. The search for coherence with what they say has led them to position themselves also on the side of the victims.

Now when there is a conflict, I have learned to pay attention to the victim who is the one who really needs my attention and support, ignoring at first the aggressor (Primary education teacher. Evidence record table).

This new environment in which they feel that, if they are attacked, they will be supported, even when they relate aggressions that have happened in other contexts, has allowed them to create bonds of trust and friendship that did not exist before. In dialogic spaces such as literary gatherings, they tell stories of violence they have experienced and which they take with them, but now the social support of the group comforts them by giving them the necessary strength to come out successfully despite having lived through difficult situations.

(…) the others support them, equal dialogue and solidarity … are present. In the gatherings, they often return to themes that always come up, to the wounds they have and the rest support and accompany them. The network of support from the rest is very important, and it creates very nice links between the students (Primary education teacher, interview).

The Zero Violence Brave Club helps them to establish relationships of more solidarity and care. The primary education students explained that now, if someone falls, they go and show concern by asking and accompanying, or if they see someone alone in the playground, they come and invite them to play with them. This type of relationship creates bonds of greater quality and trust. For this to happen, the rule of “laughing with everyone” has helped them to keep the feeling of friendship in mind.

If a child falls down … “are you OK? Can I help you?” (Primary education student 2, CFG)

playing with brave friends (Primary education student 1, CFG)

What happens if we go out in the playground and a friend is alone and brave? (Primary education teacher, CFG)

“Do you want to play with me?” (Primary education student 1, CFG)

It can be concluded that the DMPRC or the Zero Violence Brave Club as they call it, helps to create relationships of friendship that make them feel happier and safer. Protection and good treatment have become values that are taken into account when making choices about friendships. An example of this is the story of friendship that a student has managed to build up thanks to the Zero Violence Brave Club. Their teacher explained that it was unthinkable that this relationship could become a friendship because they were not capable of treating each other well. However, their story shows that when the best feelings are valued, the quality of relationships can improve.

I help Lucia (Transition to adult life program student 1, CFG)

Last year … (Secondary education teacher, CFG)

I treated her badly (Transition to adult life program student 1, CFG)

Do you remember that last year she didn’t want to be your friend because you didn’t treat her well? (Secondary education teacher, CFG)

And now good (Transition to adult life program student 1, CFG)

Now they are very good friends (Secondary education teacher, CFG)

We dance, we sing, we play music … (Transition to adult life program student 1, CFG)

It was unthinkable, that this relationship could be saved (Secondary education teacher, CFG)

**Figure 2:**
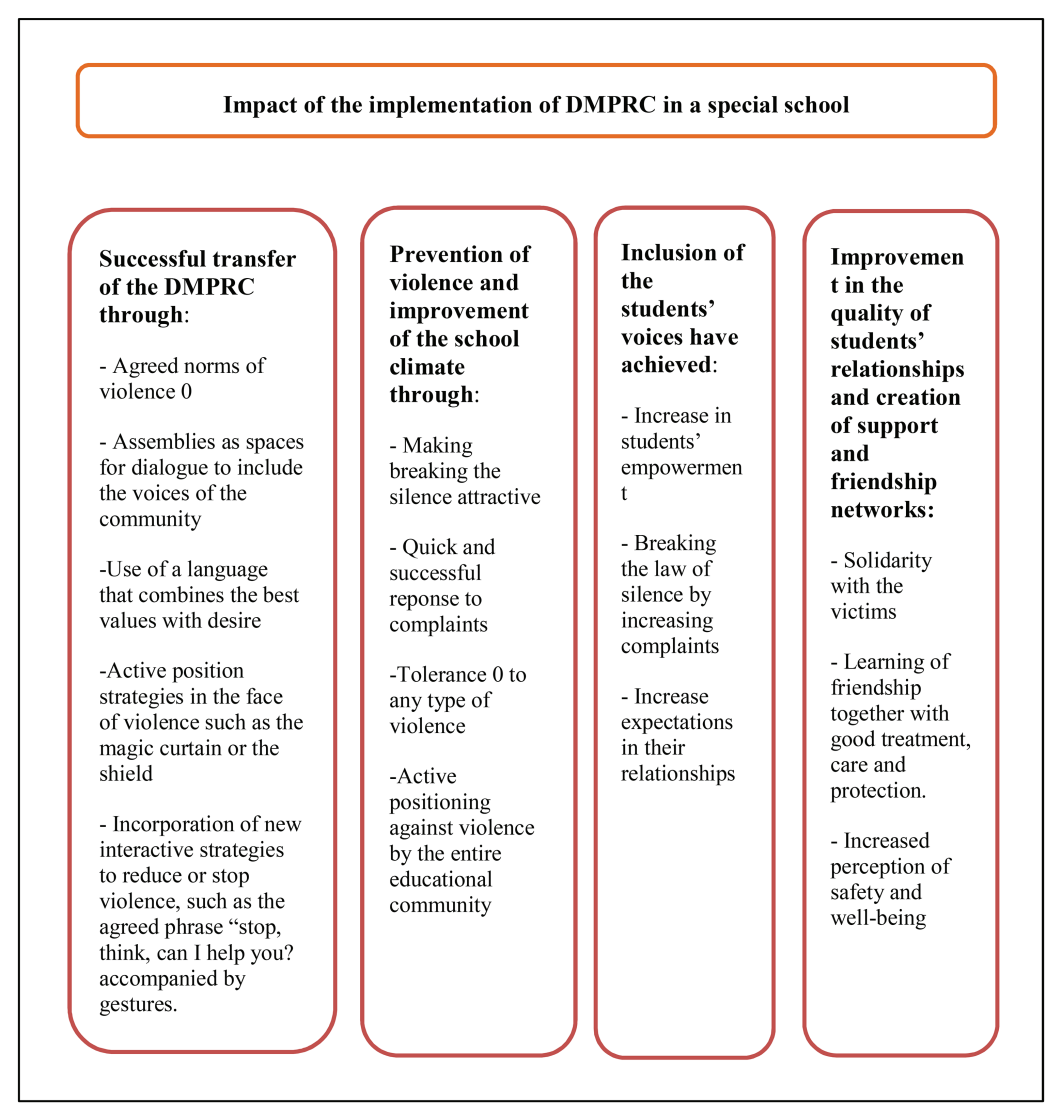
Results and impacts obtained in this study after the application of the DMPRC.

The following figure shows the different results and impacts reported in this study related to the evidence-based strategies used in the school (see Figure 2).

## Discussion

The results obtained from this study show that it is possible to transfer the DMPRC, which so far had only been implemented in mainstream schools, to a special school. The implementation of the DMPRC in a special education context overcomes barriers to participation, to the creation of support networks, and to the inclusion of the voices of students with disabilities, which left them more exposed to violence. As a result, the school climate and coexistence has been improved thanks to the preventive power of the dialogic model, and there has been an increase in the number of complaints, the empowerment of students and the creation of support and friendship networks. This has led to the creation of a safer context in which students have the confidence to report violence because teachers will listen to them, believe them, and accompany them (Banyard et al., 2010). The success of this action lies precisely in the possibility of the dialogic participation of students with special educational needs, which allows their voices not to be excluded from the creation of classroom and school rules, but rather to be present in the process from the beginning, being precisely the ones that give meaning to each of the rules.

Traditionally, there has been a tendency to approach the issues of coexistence in special schools or classrooms in a more disciplinary way and from a more behavioral perspective and not so much from a social or dialogic point of view (Beam and Mueller, 2017). The transference of the DMPRC has made it possible to create, for the first time, more dialogic and safe environments, where the voices of students with special educational needs are the protagonists, allowing these students to achieve a real participation in the creation of a policy of zero tolerance to violence in schools (UNICEF, 2015).

Some research has shown the benefits of interactive and dialogic environments for students’ learning and emotional development (García-Carrión et al., 2018). With this model of coexistence where dialogue and interactions are crucial, it becomes possible to agree on clear rules to combat violence (Eliot et al., 2010) helping to promote attitudes of active positioning against violence, even when it is not perpetrated by a peer. Students and teachers have identified that the agreed rules help them to successfully face violence, on the one hand, because these norms are known by everyone and are always reminded in different spaces and, on the other hand, because those who denounce and act against violence are socially valued by the group and have a support network that does not leave them alone.

Students with special educational needs are a vulnerable group to suffer violence (Devries et al., 2014; Malecki et al., 2020) due to the lack of social support network they have, the difficulty to relate with others, the fact that their voices tend to be unheard and the lack of active community positioning in favor of the victims (Bourke and Burgman, 2010; Rose et al., 2015; Hamby et al., 2016; Cook et al., 2017; Iotti et al., 2019; Clark et al., 2020). This model of coexistence succeeds in reducing the risk of suffering violence because it sets up support networks mobilizing the whole community in favor of the victims, works on building quality relationships and creating true friendships that protect them and make them feel safer. We know that friendships are a key protection factor in the face of bullying (Navarro et al., 2018) and that students with disabilities often have few friends (Devries et al., 2014), often becoming more isolated, which leaves them more defenseless and makes it difficult for them to report. In this sense, a key contribution of this model of coexistence is to be able to forge this network of friendship that is so valuable for students with disabilities.

Another of the barriers detected in scientific literature is the attraction to violence, which is learned from an early age, which leads to a social appreciation of those who practice it (Gómez, 2015; Navarro et al., 2018; López de Aguileta et al., 2020). With the Zero Violence Brave Club, we can see that students have begun to desire and seek relationships that exclude violence, overcoming the dominant socialization that associates desire with violence (Puigvert et al., 2019). It has been shown that the safe context and the attraction given to those who take an active stance against violence facilitates the desire to protect those who need it and favors the denounce of violence. This is because they no longer feel alone and are socially valued when they do so, gaining social status and not losing it as happens in contexts where those who tell the truth and denounce violence are labeled snitches (Mayes et al., 2003). The feeling of belonging to a classroom and school has increased, and this union enables them to be more courageous and gives them the strength to reject those who behave violently. The collective dream that everyone should be brave, that is, treat each other well, show solidarity and make each other feel happier, is present in the voices of the students and teachers. The DMPRC has made it possible to create this collective dream in the imagination of the community and to take steps to make it a reality.

Some authors define a positive school climate when students feel safe, have loving and caring relationships with their peers and with adults, have a sense of belonging to the school, participate meaningfully in school policies, disapprove risky behavior among their peers, and feel that their peers care about them (Cohen et al., 2009; Osher et al., 2012). The results show that students and teachers perceive that the school climate has improved and acknowledge that they feel better and happier. They associate it with the possibility of their voices counting as for example when they are delegates in school assemblies and represent their classrooms or with the empowerment they experience and allows them to publicly disapprove of aggressive behavior from their peers. We have already seen that they have also improved their relationships with their peers and with teachers thanks to the more dialogic and egalitarian interactions they are experiencing.

This is the first time that the DMPRC has been transferred to a special school and it has been possible to carry it out in the same way as in mainstream schools. As in regular schools, they have incorporated strategies that have helped them to put into practice active positioning or protective nets, such as “curtain or shield making.” But in this school, and due to the characteristics of the students, most of whom have communication and self-control problems due to their disability, new strategies have been integrated which have helped them to stop cowardly attitudes (not treating well, violence, lack of respect) and to anticipate violent behavior by stopping it before it appears, or if it does occur, preventing it from escalating. This finding can be very useful for teachers in other special schools or teachers who deal with special education students with behavioral problems, as evidence shows that a large part of teachers do not have strategies to successfully deal with this type of behavior (Stevenson et al., 2020). Some of the strategies that have been effective for them are songs or the phrase “think, talk, can I help you?” together with gestures that have been agreed upon. This strategy has made it easier for the child who is about to be violent to transform the aggressive behavior into a more prosocial one thanks to the interaction with his peers (Villardón-Gallego et al., 2018). This new contribution could enrich this successful performance in other educational settings. It has been shown that when there is a positive climate, students are less likely to bully, among other reasons because children are more likely to report violence if they witness it and more likely to seek help if they are victims (Howe et al., 2013). The results show that the MPDC is succeeding in improving the climate and increasing the number of reports. These findings show the effectiveness of this model of coexistence in preventing violence, stopping it before it appears or in its early stages.

Although this communitarian and dialogic model has given students greater self-confidence, leading them for the first time to ask to be heard when they have seen or suffered an aggression, they recognize that in other spaces their voices are still not as heard or valued. The impact outside the school has been on the closest circles, such as siblings, mothers, and fathers, thanks to the possibility of participating in community meetings or in dialogic spaces such as literary gatherings. The participation of the family in the dialogic spaces has been detected as a key issue to facilitate the transference of the results to other contexts out of school. The challenge would be to achieve a greater participation of diverse people so that other spaces in which these students participate would be transformed into safer spaces where active positioning against violence would be valued as it is in the school.

The language of possibility that is present in the DMPRC makes it possible to overcome the language of difficulty that these students normally face. The possibility of being listened to, of demanding to be treated well, of talking about the violent situations they experience and of asking for support appears clearly in their lives for the first time. The possibility also arises of having quality friendship relationships which, for students with disabilities, are very important due to the benefits they have for health and happiness according to the largest longitudinal study on the topic (Harvard Study of Adult Development). A whole world of possibilities, freedom, and happiness is opened up to them, of which they had been set apart by the fact of having a disability. These students have moved from being potential victims to being leaders of change, and this empowers them to be able, little by little, to transform other contexts. By becoming leaders of social change, they can imagine themselves as children who take a stand against violence, reject it, and be an example to others. Finally, feeling more satisfied, happier and with a certain sense of deciding more freely and rationally about their relationships and their lives could have a long-term impact on improving how they perceive themselves and their health in adulthood (Shah et al., 2014). Given that many people with disabilities are associated with health problems, a potential area of research opens up on the impact of the DMPRC on improving the health of children with disabilities.

Finally, we see two challenges. The first one would be to analyze in greater depth how, through the work with families and the inclusion of their voices, the DMPRC could have a greater impact outside the school context, since the interviews carried out have shown some barriers to the transferability of the DMPRC to other contexts where this type of students interact. The second challenge would be to study more in-depth how a profound transformation in the desire for non-violence is being generated, as it is a challenge to obtain lasting evidence on this topic with children with disabilities.

## Data Availability Statement

The raw data supporting the conclusions of this article will be made available by the authors, without undue reservation.

## Author Contributions

ED and ER-C conceived the idea of the study. ER-C conducted the fieldwork. SC, ED, and LB contributed to the literature review. SC wrote a first draft of the paper with the support of ER-C. LB and ED conducted a review of the draft and provided feedback. SC included the feedback and wrote the final version of the manuscript. All authors have read and agreed to the submitted version of the manuscript.

### Conflict of Interest

The authors declare that the research was conducted in the absence of any commercial or financial relationships that could be construed as a potential conflict of interest.

## Footnotes

^1^She refers to the seven principles of the dialogic learning: egalitarian dialogue, cultural intelligence, transformation, instrumental dimension, creation of meaning, solidarity, and equality of differences (Flecha, 2000).
